# Diagnostic Accuracy of Different Soluble fms-Like Tyrosine Kinase 1 and Placental Growth Factor Cut-Off Values in the Assessment of Preterm and Term Preeclampsia: A Gestational Age Matched Case-Control Study

**DOI:** 10.3389/fmed.2018.00325

**Published:** 2018-11-30

**Authors:** Evelyn A. Huhn, Andrea Kreienbühl, Ina Hoffmann, Andreas Schoetzau, Soeren Lange, Begona Martinez de Tejada, Martin Hund, Irene Hoesli, Olav Lapaire

**Affiliations:** ^1^Department of Obstetrics and Gynecology, University Hospital Basel, Basel, Switzerland; ^2^Department of Obstetrics and Gynecology, Institutions Hospital du Nord Vaudois, Yverdon-les-Bains, Switzerland; ^3^Department of Obstetrics and Gynecology, Geneva University Hospitals and Faculty of Medicine, University of Geneva, Geneva, Switzerland; ^4^Roche Diagnostics International, Rotkreuz, Switzerland

**Keywords:** preeclampsia, hypertension in pregnancy, diagnosis, biomarker, sFlt-1, PlGF, sFlt-1:PlGF ratio

## Abstract

**Introduction:** The objective was to investigate the diagnostic accuracy of different thresholds of the soluble vascular endothelial growth factor receptor-1 (sFlt-1) and the placental growth factor (PlGF) in preterm (≤37 weeks) and term (>37 weeks) preeclampsia (PE).

**Materials and Methods:** A nested case-control study was performed from a high-risk Swiss cohort. Only blood samples on the day of PE diagnosis were included. The primary outcome was to verify the diagnosis using the recently proposed cut-off values for PE (sFlt-1:PlGF ratio of ≥85 in ≤ 34 weeks or ≥110 in >34 weeks), and the gestational age dependent centiles.

**Results:** Thirty-four women with preterm PE were matched with 64 controls and 25 women with term PE with 45 controls. The test performance of the sFlt-1:PlGF ratio in preterm PE was very good (AUROCC of 0.95). The sFlt-1:PlGF ratio could adequately predict adverse fetal or neonatal outcome. In term PE, sFlt-1 alone showed a slightly better diagnostic accuracy with an AUROCC of 0.84. Almost all women with a sFlt-1:PlGF ratio above threshold delivered during the following week.

**Discussion:** In pregnant women with high risk of developing PE, the sFlt-1:PlGF ratio and sFlt-1 levels help clinicians to confirm the diagnosis of imminent preterm PE and can additionally be used to rule out PE at term.

## Introduction

Preeclampsia (PE) affects around 3–5% of pregnancies (1) and is still, together with other hypertensive disorders in pregnancy, the second most common direct cause of maternal mortality (14% of all maternal deaths) worldwide (2). Four of five PE events occur >37 weeks of gestation (WOG) (3). Term PE is less often associated with placental dysfunction and the fetus is therefore less threatened with intrauterine growth restriction (IUGR) as in preterm PE. But term PE is by no means a benign condition. Severe fetal and maternal complications can occur. Twenty percent of the cases with HELLP (haemolysis, elevated liver enzymes and low platelets) syndrome (4) and 55% of the cases with eclampsia develop in term PE (5).

As clinical diagnosis dependent on maternal signs and symptoms can be ambiguous, recent research has focused on circulating biomarkers in maternal blood to improve the assessment of PE (6). The proangiogenic biomarker, placental growth factor (PlGF), and the antiangiogenic biomarker, soluble vascular endothelial growth factor receptor-1 (sFlt-1), both mainly derived from the placental trophoblast, seem to accurately diagnose and predict early onset preeclampsia (delivery ≤ 34 WOG) (7) or even preterm PE (PE ≤ 37 WOG) as well as fetal and neonatal adverse outcomes (8) and might improve the allocation of care (9, 10). Normally, sFlt-1 begins to rise after 30–32 WOG and PlGF starts to fall after 30 WOG (11). These biochemical changes seem to result to some extent from cellular stress in the syncytiotrophoblast, which occurs during the last 8–10 weeks of a normal pregnancy (12). If the normal and abnormal states cannot be distinguished clearly, the diagnosis of term PE can be challenging—especially when the PlGF and sFlt-1 levels before the onset of symptoms are unknown and when other medical conditions and risk factors are already present, such as pre-existing or gestational hypertension.

The aim of this study was to evaluate the diagnostic accuracy of the recently proposed GA dependent centiles for PlGF, sFlt-1, and sFlt-1:PlGF ratio, and the simplified cut-off values for sFlt-1:PlGF ratio (13) in assessing the diagnosis of preterm and term PE vs. a high risk control group.

## Materials and methods

We performed a nested case-control study among a high-risk cohort recruited at the University Hospitals of Basel and Geneva, Switzerland. The Competent Ethics Committee of North-western Switzerland and of Geneva (EKBB 251/11 and GE 14-216) approved the study protocol and written informed consent was obtained from all participating women.

Women who were ≥18 years of age with a singleton pregnancy were considered for enrolment if they had at least one risk factor for PE: Nulliparous overweight or obese women with body mass index (BMI) ≥26.1 kg/m^2^, nulliparous women >40 years of age, pre-existing diabetes, essential hypertension or renal disease, pregnancy induced hypertension, gestational diabetes mellitus (defined by at least one pathological value of fasting glucose (≥5.1 mmol/l) or at one (≥10.0 mmol/l) or 2 h (≥8.5 mmol/l) after 75 g glucose load), utero-placental dysfunction (defined by abnormal uterine perfusion with mean pulsatility index >95th percentile in the second trimester and/or bilateral uterine artery notching), previous PE, eclampsia or HELLP (haemolysis, elevated liver enzymes, and low platelets) syndrome, thrombophilia with high risk for PE (homozygous factor V Leiden or methylenetetrahydrofolate reductase (MTHFR) C677T defects, or the combination of heterozygous factor II G20210A and heterozygous factor V Leiden defects diagnosed in a DNA analysis prior pregnancy), antiphospholipid antibodies and family history of PE, eclampsia or HELLP syndrome in first line relatives. Additionally, women who had symptoms suspicious of PE [two combined findings of clinical symptoms like headache and/or scotoma and/or epigastric pain and/or excessive edema and/or new onset proteinuria (≥1+ in dipstick)] were asked to participate. Exclusion criteria were chromosomal aberrations, fetal malformations, abortion or stillbirth < 22 WOG. All eligible women were informed about the study during antenatal visits or during hospitalization between 15 and 42 WOG until delivery and followed regularly, with demographic characteristics, medical history, clinical examinations, and blood drawings for biomarker analysis (PlGF, sFlt-1) documented. High risk women with clinical findings suggestive of PE and symptomatic women were followed clinically and biochemically over a period of 1 to 14 days depending on their conditions until delivery. Only patients with blood sampling on the day of the PE diagnosis were included for this case-control study.

### Diagnostic criteria for hypertensive diseases in pregnancy

Pre-existing hypertension was defined as systolic blood pressure ≥140 mmHg and/or diastolic blood pressure ≥90 mmHg diagnosed before conception or ≤ 20 weeks of gestation. Gestational hypertension was determined as new onset of hypertension developing >20 weeks of gestation without proteinuria.

The “traditional” criteria for PE were used to establish the diagnosis: (14, 15) New onset systolic blood pressure ≥140 mmHg and/or diastolic blood pressure ≥90 mmHg measured on two occasions at least 6 h apart but within 1 week and new onset proteinuria with ≥300 mg/24h urine protein collection or ≥2+ in dipstick or spot urine (≥30 mg/dL or protein/creatinine ratio ≥30 mg protein/mmol creatinine) >20 WOG. Preterm PE was defined as the presence of PE ≤ 37 WOG and term PE as the development of PE >37 WOG. Eclampsia was defined as new onset of tonic-clonic seizures associated with PE, which could not be assigned to any other cause. HELLP syndrome was considered when haemolysis (lactic acid dehydrogenase >600 IU/L, and/or lowered haptoglobin), elevated liver enzymes (aspartate amino transferase exceeding 70 IU/L) and low platelets (platelet counts < 100,000/μL) occurred.

Severe PE was defined as systolic blood pressure ≥160 mmHg and/or diastolic blood pressure ≥110 mmHg and/or the presence of HELLP syndrome and/or creatinine level of ≥99 mmol/L and/or pulmonary oedema and/or severe cerebral/visual symptoms (16, 17).

### Diagnostic criteria for fetal, neonatal, and maternal adverse events

Intrauterine growth restriction (IUGR) was defined as an estimated fetal weight ≤ 10th percentile (adjusted for gender and ethnicity according to charts routinely used by both sites) plus pathological finding(s) in the Doppler indices (cerebro-placental ratio ≤ 5th percentile and/or pulsatiliy index of uterine arteries ≥95th percentile in second trimester) or a birth weight ≤ 3rd percentile (18). Maternal adverse events were defined as follows: Pulmonary oedema, acute renal injury (increase in serum creatinine by ≥0.3 mg/dl (≥26.5 umol/l) within 48 h or increase in serum creatinine of ≥1,5 times baseline within prior 7 days or urine volume < 0.5 ml/kg/h for 6 h), cerebral hemorrhage, disseminated intravascular coagulation or death. The fetal adverse events were defined as follows: Perinatal or neonatal death up to 6 weeks after delivery, preterm birth ≤ 34 WOG, IUGR, placental abruption, respiratory distress syndrome, necrotising enterocolitis, or intraventricular hemorrhage (8).

### Assessment of PlGF and sFlt-1 and sFlt-1:PlGF cut-off values

All maternal serum samples were aliquoted and stored at −80°C until measurement of sFlt-1 and PlGF. All samples had not been thawed before the day of measurement. Serum levels of sFlt-1 and PlGF were determined using the Roche Elecsys® sFlt-1 and Elecsys® PlGF assays on the electrochemiluminescence immunoassay platforms, Modular® E170 (Roche Diagnostics GmbH, Mannheim, Germany) until October 2014 and Cobas® 6000 (Roche Diagnostics GmbH, Mannheim, Germany) from November 2014 until the end of study. The within-run coefficient of variation for quality control samples was below 1.5% for the sFlt-1 and below 0.9% for the PlGF assay on Modular® E170. Between-run coefficients of variation were 2.5 to 3.9% for the sFlt-1 and 2.7 to 3.7% for the PlGF assay on Modular® E170 and 1.2 to 2.3% for the sFlt-1 and 1.7 to 2.0% for the PlGF assay on the Cobas® 6000 platform. Results of the biomarker analysis were not available until the end of study and could not influence management decisions.

Firstly, the simplified cut-off values with ≥85 for ≤ 34 WOG and ≥110 for >34 WOG for the sFlt-1: PlFG ratio were tested (9, 13). In addition, for the GA-dependent centile values, the 5th and the 95th centile of the normal distribution for the single biomarkers PlGF and sFlt-1 as well as the sFlt-1: PlGF ratio (13) were used in preterm and term PE vs. the GA-matched high-risk control group.

### Statistics

The total enrolment target was calculated by sample size calculation before the beginning of the study. The prevalence of PE was assumed to be around 15% in the high risk (50% contribution) and 20% in the symptomatic subgroup (50% contribution). The calculation was performed with a proposed true area under the receiver operating characteristic curve (AUROCC) of 0.92 for the predictive test performance of single biomarkers with a lower boundary of 0.8 (95% CI > 0.8), a power of 80%, and an α-level of 5% and an estimated drop out of 15% (*n* = 251).

Baseline characteristics were stratified for women with preterm and term PE and the GA-matched (± 4 days) risk women. Continuous variables were tested for differences between the groups using Wilcoxon signed-rank tests and chi square tests for categorical variables. We estimated and compared the diagnostic accuracy [sensitivity, specificity with their 95% Confidence Interval (CI)] using the proposed cut-off values of ≥85 for ≤ 34 WOG and of ≥110 for >34 WOG and the GA dependent cut off values for the single biomarkers PlGF and sFlt-1 for the detection of PE according to Verlohren et al. (13). ROC curves were presented with the corresponding AUROCC and corresponding 95% CI for PE diagnosis. A level of significance of *P* < 0.05 was used. All statistical analyses were performed with the statistical software R version 3.3.1 (19).

## Results

### Baseline characteristics

**Figure 1:**
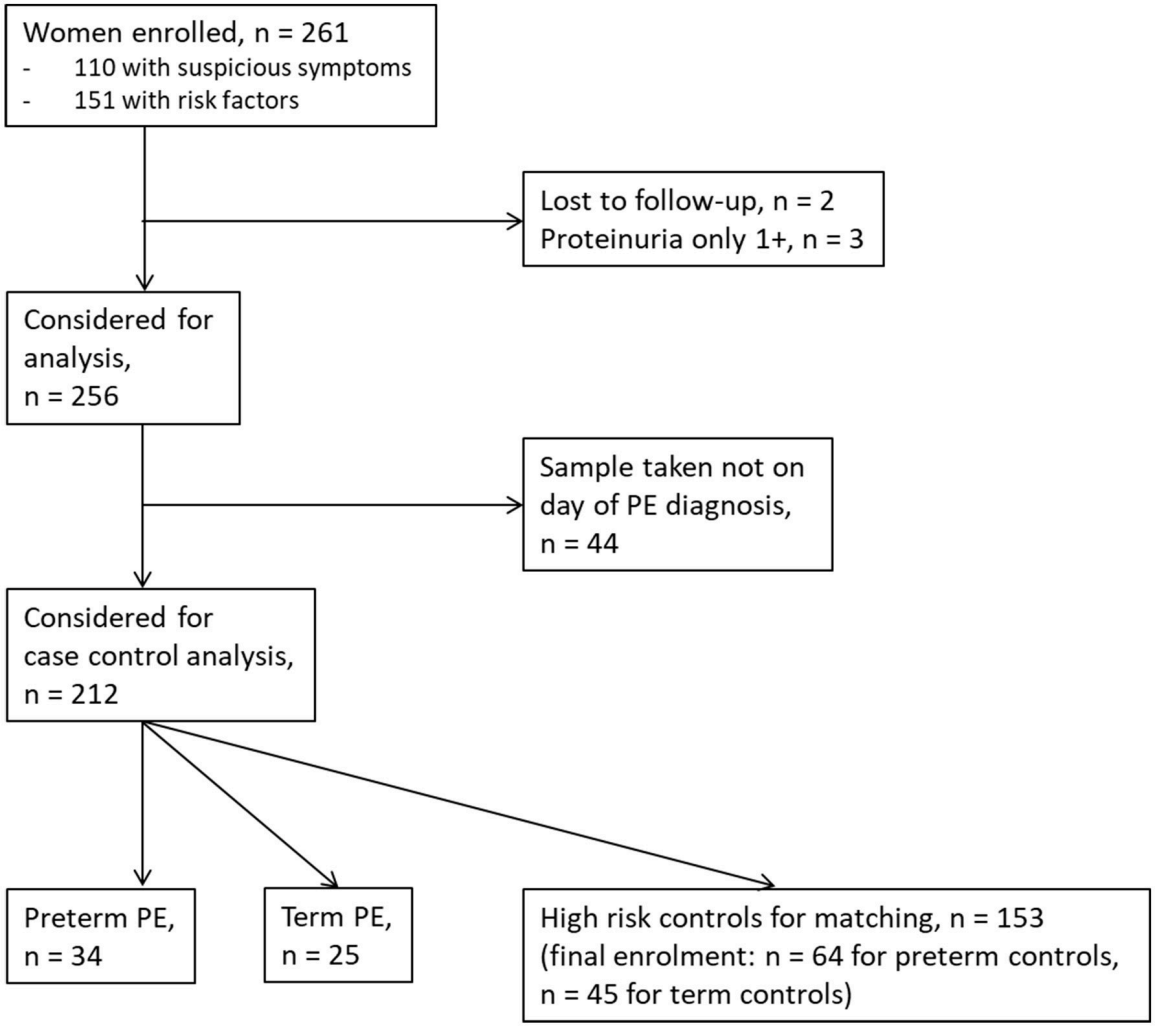
Flow chart of enrolments and exclusions.

**Figure 2:**
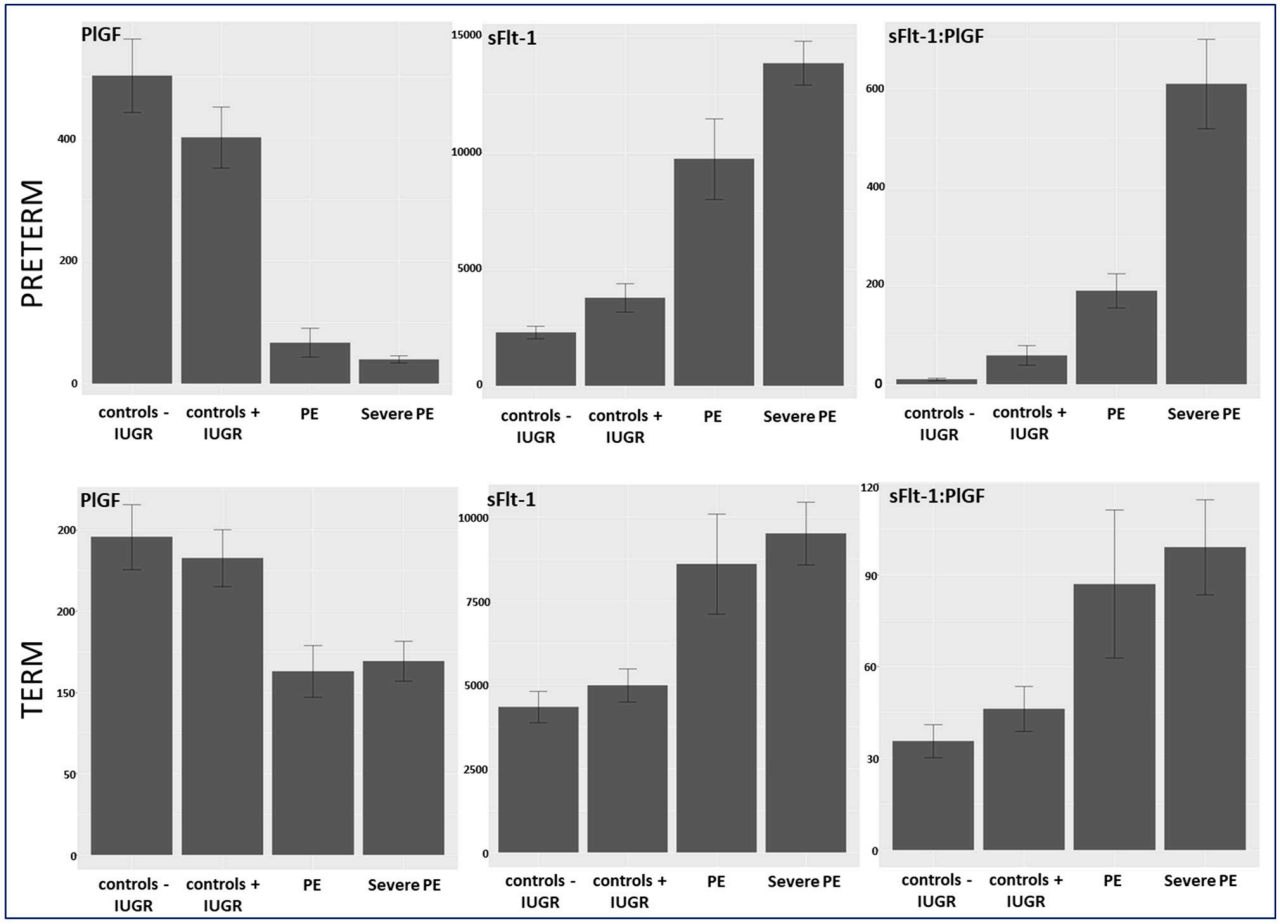
Biomarker levels of PlGF, sFlt-1, and sFlt-1:PlGF ratio stratified in controls –/+ IUGR, PE, and severe PE in preterm and term group. IUGR, intrauterine growth restriction; PE, preeclampsia; PlGF, placental growth factor; sFlt-1, soluble vascular endothelial growth factor receptor-1.

**Figure 3:**
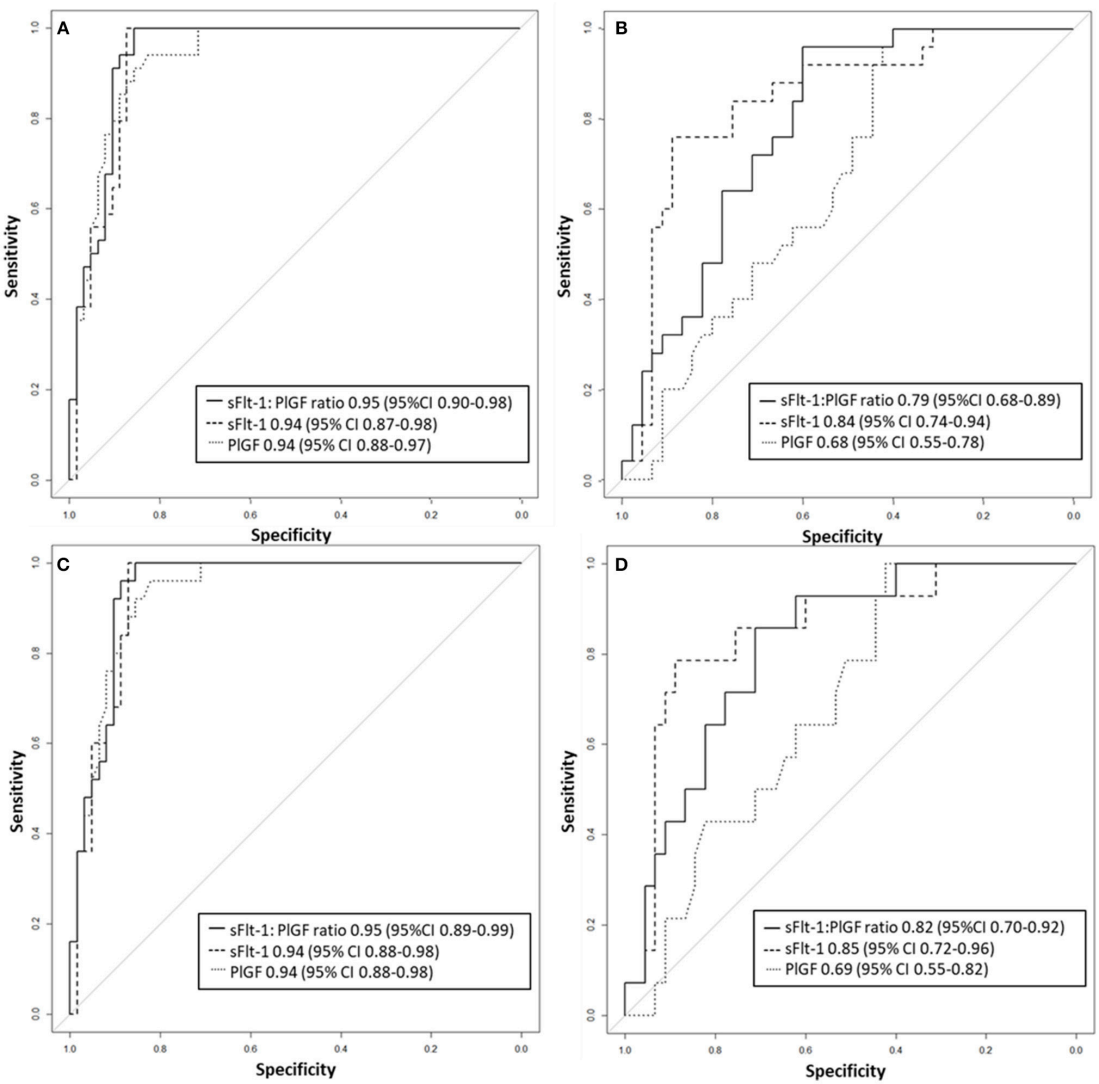
Receiver operator characteristic curve analysis for sFlt-1:PlGF ratio and single biomarkers for the detection of **(A)** preterm PE and **(B)** term PE as well as **(C)** severe preterm, and **(D)** severe term PE. PE, preeclampsia; PlGF, placental growth factor; sFlt-1, soluble vascular endothelial growth factor receptor-1.

**Figure 4:**
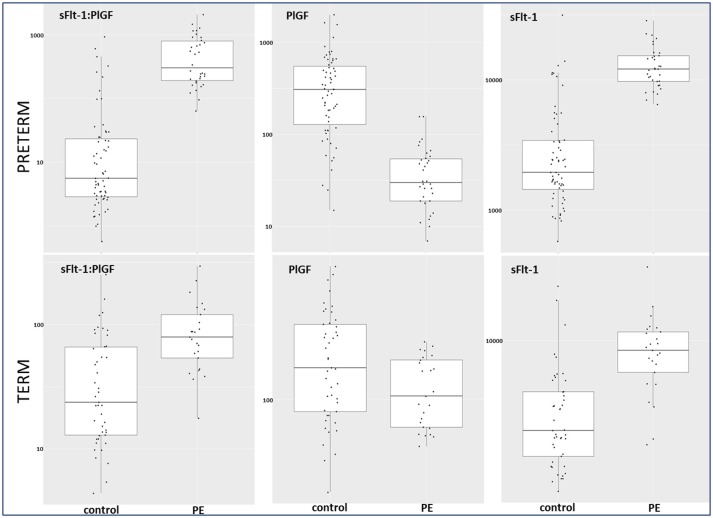
Box plots for sFlt-1:PlGF, PlGF, and sFlt-1 in preterm and term control and PE groups. PE, preeclampsia; PlGF, placental growth factor; sFlt-1, soluble vascular endothelial growth factor receptor-1.

**Figure 5:**
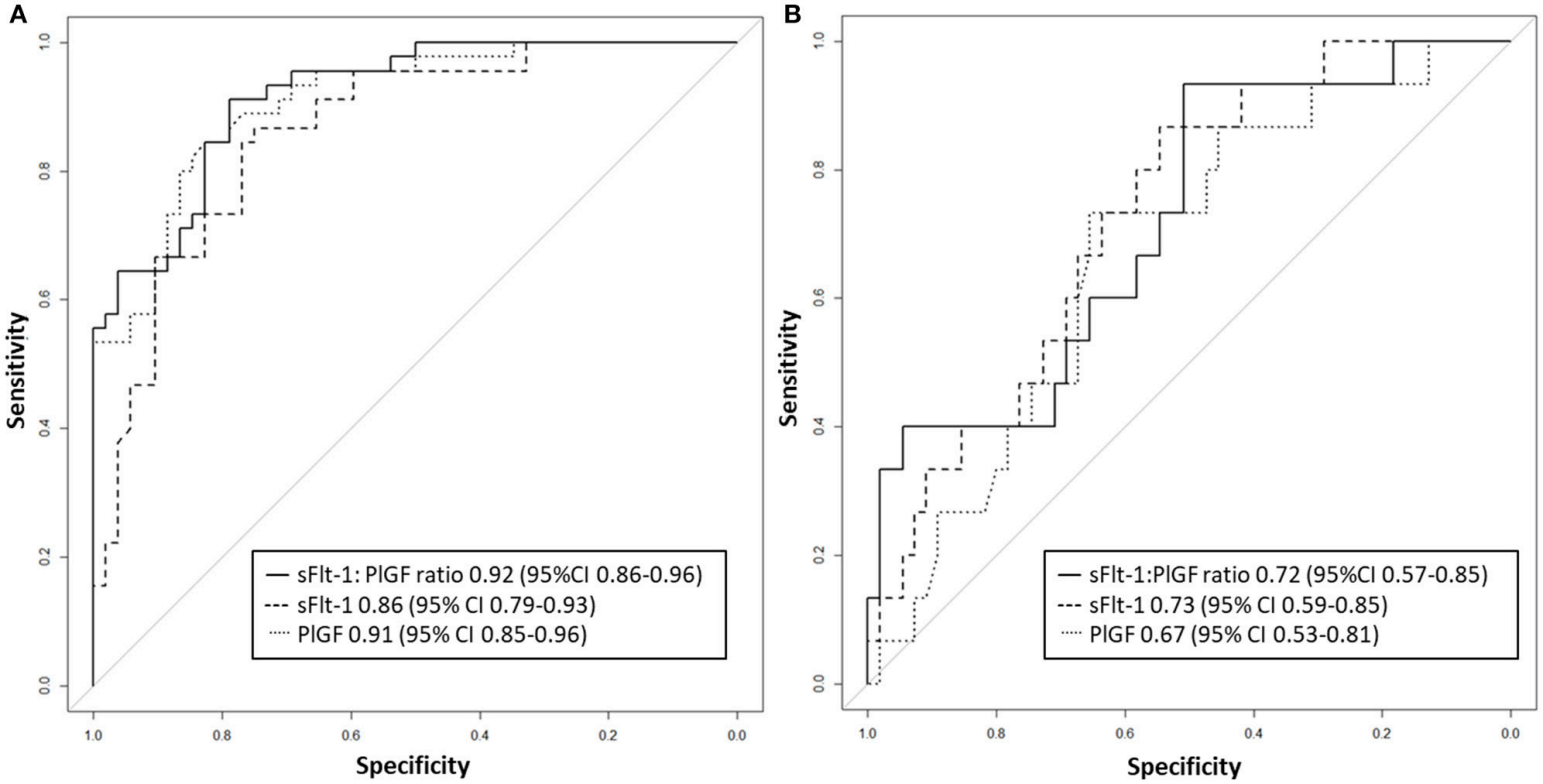
Receiver operator characteristic curve analysis for sFlt-1:PlGF ratio and single biomarkers for the detection of fetal/neonatal adverse events in **(A)** preterm and **(B)** term groups. PlGF, placental growth factor; sFlt-1, soluble vascular endothelial growth factor receptor-1.

**Figure 6:**
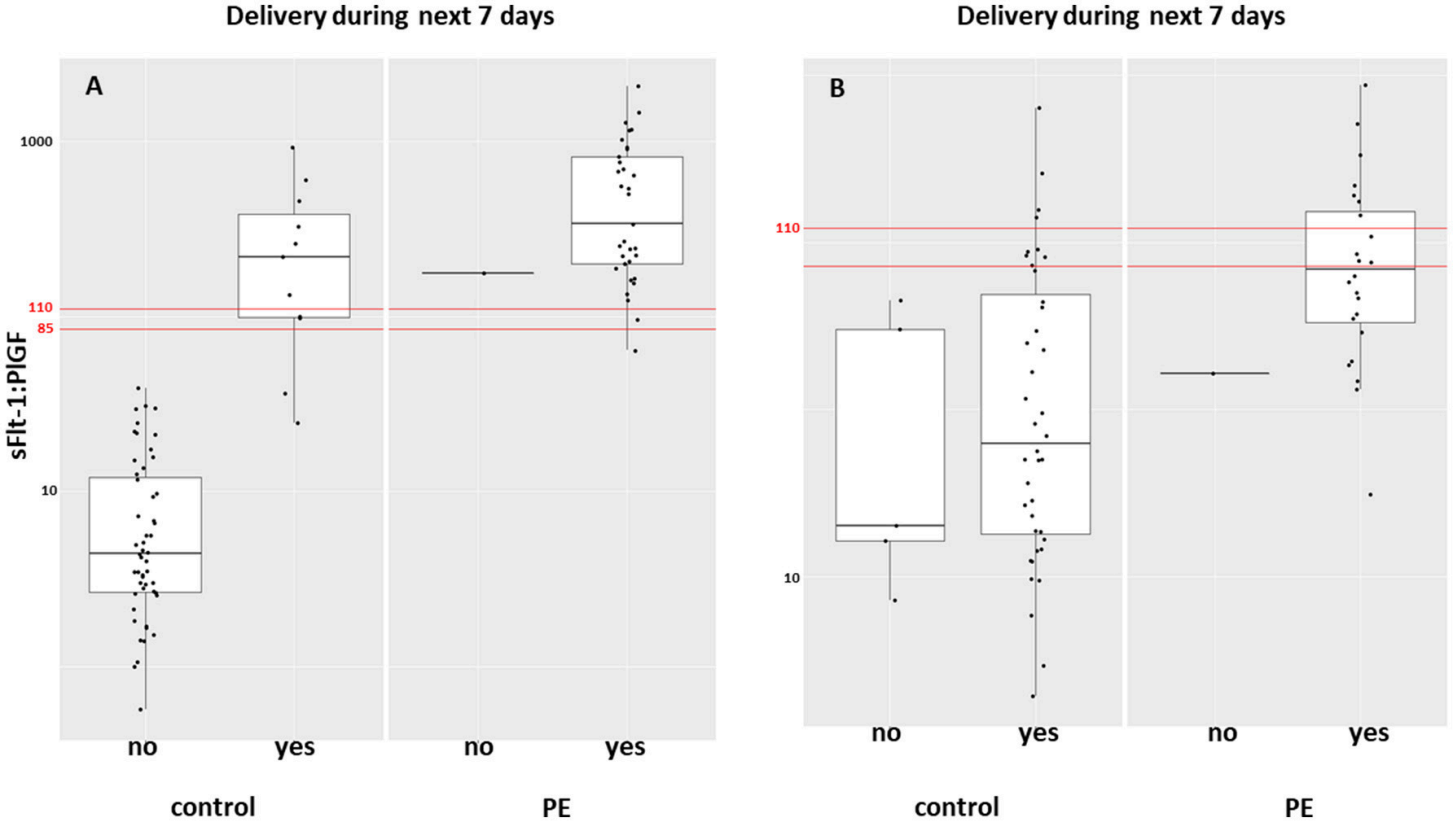
Box plot of the sFlt-1:PlGF ratio in women with PE and controls who delivered during the following week in **(A)** preterm and **(B)** term groups. PE, preeclampsia; PlGF, placental growth factor; sFlt-1, soluble vascular endothelial growth factor receptor-1.

Between September 2011 and July 2015, a total of 261 women were recruited for this prospective cohort study. Thirty-four women with preterm PE were matched for GA with 64 controls and 25 women with term PE with GA-matched 45 controls for a nested case-control approach (see Figure 1 for a flow chart of recruitments and exclusions). The maternal characteristics and the risk constellation of all groups are summarized in Table 1. The maternal characteristics and the risk constellation were not statistically significant apart from having more nulliparous women in the term PE than in the term control group (*p* = 0.047). Table 2 summarizes the delivery characteristics and the maternal and fetal/neonatal adverse events. The number of IUGRs was high both in control and PE groups, but did not differ significantly between groups [preterm: 15 (23.4%) vs. 11 (32.4%); term: 7 (16.3%) vs. 4 (17.4%)]. The median GA at time of blood collection was 30+3 WOG in the preterm PE and 30+6 WOG in the control group and 38+5 WOG in both term groups, but the preterm PE group had a significantly shorter interval between blood sampling and delivery [PE: 1 day (d) (Interquartile range (IQR), 0–11 d) vs. control group: 33 d (IQR, 0–124 d), *P* < 0.001]. There were no differences in the interval between blood sampling and delivery in both term groups [PE: 1 d (IQR, 0–4 d) vs. control group: 0 d (IQR, 0–25 d)]. In the preterm PE group, the median of the sFlt-1:PlGF ratio was 304 [IQR, 63.9–2075]. The preterm control group had a low sFlt-1:PlGF ratio of 5.4 [IQR, 0.6–927]. The high upper quartile was due to pregnancies complicated by severe IUGRs in the control group which were delivered due to fetal indications (spontaneous decelerations in the cardiotocogram, low short term variation in the computerized cardiotocogram or pathological Doppler values). Thirteen of 15 IUGR pregnancies had no sign of hypertension, 1 pregnancy which was complicated by IUGR had also pre-existing hypertension and 1 pregnancy with IUGR had mild *de-novo* hypertension (with a sFlt-1:PlGF ratio of 30). Figure 2 shows the PlGF and sFlt-1 levels and the sFlt-1:PlGF ratio stratified for PE, severe PE and controls with and without accompanying IUGR. The term PE and its control groups had generally lower sFlt-1:PlGF ratios with 79.3 [IQR, 17.6–296] and 23.7 [IQR, 4.39–252], (*P* < 0.001), higher sFlt-1 levels of 8999 [IQR, 3232–22212] and 3770 [IQR, 1953–18050] (*P* < 0.001) and lower PlGF levels of 104 [IQR, 56–202] in PE vs. 147 [IQR, 32.0–511] (*p* = 0.03) in the control group vs. the preterm groups. Figure 3 shows the ROC curves for the ratio and the single biomarkers in preterm and term groups to assess PE and severe PE. In preterm PE, the sFlt-1:PlGF ratio, PlGF, and sFlt-1 and show similar performance [AUROCC of sFlt-1:PlGF ratio 0.95 (95% CI, 0.90–0.98), PlGF 0.94 (95% CI, 0.88–0.97) and sFlt-1 0.94 (95% CI, 0.87–0.98)] and in term PE, sFlt-1 seems to be slightly better in detecting PE with an AUROCC of 0.84 (95% CI, 0.74–0.94) vs. the sFlt-1:PlGF ratio [0.79 (95% CI, 0.68–0.89)]and PlGF alone [0.68 (95% CI, 0.55–0.78)]. The boxplots in Figure 4 show a slight trend toward better discrimination between women with PE and high risk using sFlt-1 alone than the sFlt-1:PlGF ratio or PlGF, but CIs of single biomarkers and the ratio overlap widely. The highest sensitivity with 0.48 and a specificity of 0.93 is reached if sFlt-1 alone was used with a single cut off value ≥9,184 (= 95th centile) at >37 WOG. All results regarding diagnostic accuracy for the simplified and the GA dependent centile values of the ratio and the single biomarkers are presented in Table 3. The single biomarkers PlGF and sFlt-1 and the sFlt-1:PlGF ratio demonstrated good specificity for ruling out term PE (0.93 for single biomarkers and 0.91, respectively). Only 14 women in the term PE group showed signs of severe PE. The 95th centile of sFlt-1 showed a similar AUROCC of 0.85 (95% CI, 0.72–0.96) for the assessment of severe PE. Figure 5 shows the ROC curve analysis for the detection of any fetal/neonatal adverse events in the preterm and term groups. In preterm PE, PlGF, sFlt-1, and sFlt-1:PlGF ratio show good performance [AUROCC of PlGF 0.91 (95% CI, 0.85–0.96), sFlt-1 0.86 (95% CI, 0.79–0.93) and sFlt-1:PlGF ratio 0.92 (95% CI, 0.86–0.96)]. In term groups, the number of fetal adverse events was low (*n* = 8). SFlt-1 performed with an AUROCC of 0.72 (95% CI, 0.57–0.85), PlGF with 0.67 (95% CI, 0.53–0.81) and the sFlt-1:PlGF ratio with 0.72 (95% CI, 0.57–0.85), respectively. Almost all PE and control women with a sFlt-1:PlGF ratio above the threshold of ≥85 or ≥110 in preterm (38/39) and ≥110 in the term group (11/11) delivered during the following week whether or not they developed PE (see box plots of sFlt-1:PlGF ratio in Figure 6). Thirty-one women of the 38 preterm pregnancies developed PE and the other 7 women had pregnancies complicated by IUGR, which were delivered due to fetal indications. In the term groups, 7 of the 11 pregnancies with a ratio ≥110 which delivered within a week developed PE, 3 were complicated by IUGR and only one had no IUGR but a pregnancy with gestational diabetes mellitus and delivered within a week.

**Table 1:** Maternal characteristics and risk constellation.

**Characteristics**	**Preterm PE *n* = 34**	**Control *n* = 64**	***p*-value**	**Term PE *n* = 25**	**Control *n* = 45**	***p*-value**
Age, yr	31.0 [18.0; 44.0]	32.0 [18.0; 48.0]	n.s.	30.0 [24.0; 39.0]	31.0 [20.0; 45.0]	n.s.
BMI (current),kg/m ^2^	30.6 [21.6; 45.5]	31.1 [21.3; 57.8]	n.s.	31.1 [25.2; 57.6]	30.4 [21.7; 51.7]	n.s.
Ethnicity			n.s.			n.s.
- Asian	2 (5.9)	6 (9.4)		0	2 (4.4)	
- Black	2 (5.9)	0		0	2 (4.4)	
- Caucasian	30 (88.2)	57 (89.1)		22 (95.7)	41 (91.1)	
- Others	0	1 (1.6)		1 (4.4)	0	
Parity	0.00 [0.00; 4.00]	0.00 [0.00; 3.00]	n.s.	0.00 [0.00; 1.00]	0.00 [0.00; 3.00]	0.047
Primigravida	23 (67.6)	35 (55.6)	n.s.	18 (72)	23 (51.1)	n.s.
Risk constellation (multiple selections possible)						
- nulliparous women and age ≥40yr	2 (5.9)	3 (4.7)	n.s.	0	3 (6.7)	n.s
- nulliparous women and BMI						
prepreg. > 26,1	1 (2.9)	4 (6.3)	n.s.	0	3 (6.7)	n.s.
- previous PE	4 (11.8)	16 (25)	n.s.	3 (12)	14 (31.1)	n.s.
- family history of PE	1 (2.94)	1 (3.7)	n.s.	0	0	n.s.
- preexisting hypertension	8 (23.5)	13 (21)	n.s.	3 (12)	8 (16.8)	n.s.
- gestational hypertension	6 (18.2)	7 (10.9)	n.s.	4 (16)	8 (16.8)	n.s.
- nephropathy	3 (8.8)	4 (6.3)	n.s.	0	3 (6.7)	n.s.
- preexisting diabetes	0	2 (3.1)	n.s.	0	2 (4.4)	n.s.
- gestational diabetes	2 (5.9)	13 (20.3)	n.s.	4 (16)	17 (38.6)	n.s.
- thrombophilia/anti- phospholipid antibodies	2 (5.9)	2 (3.1)	n.s.	0	0	n.s.
- abnormal uterine artery	10 (30.3), *n* = 33	8 (13.3), *n* = 60	n.s.	0, *n* = 25	O, *n* = 40	n.s.
Doppler					

*Reported are median and interquartile range or number and percentage BMI, body mass index; PE, preeclampsia; yr, year*.

**Table 2:** Delivery characteristics and maternal and fetal/neonatal adverse events.

**Characteristic**	**Preterm PE *n* = 34**	**Control *n* = 64**	***p*-value**	**Term PE *n* = 25**	**Control *n* = 45**	***p*-value**
GA at delivery, wk+d	30+4 [22+0; 37+3]	38+3 [26+3; 42+0]	< 0.001	39+0 [37+4; 41+4]	39+4 [37+0; 41+4]	n.s.
Maternal adverse events			< 0.001			n.s.
- Eclampsia	1 (2.9)	0		0	0	
- HELLP syndrome	2 (5.9)	0		2 (8)	0	
- Death	0	0		0	0	
- Cerebral hemorrhage	0	0		0	0	
- Cerebral thrombosis	0	0		0	0	
- Pulmonary oedema	1 (2.9)	0		0	0	
- Acute renal injury	1 (2.9)	0		0	1 (2.2)	
- DIC	0	0		0	0	
Birth weight,g	1295 [400; 2770]	2950 [576; 4280]	< 0.001	3150 [2360; 4220]	3280 [1690; 4740]	n.s.
Fetal/neonatal adverse events (multiple selections possible)			< 0.001^**^			n.s.^**^
- Fetal or perinatal death	1 (2.9)	1 (1.6)	n.s.	0	0	
- Preterm birth < 34	15 (44.1)	3 (4.7)	0.05	NA	NA	
WOG						
- IUGR	11 (32.4)	15 (23.4)	n.s.	4 (16)	8 (17.8)	
- Placental abruption	1 (2.9)	1 (1.6)	n.s.	0	0	
- ARDS	13 (38.2)	6 (9.4)	0.004	3 (12)	0	
- NEC	1 (2.9)	1 (1.6)	n.s	0	0	
- IVH			n.s			
IVH 1-11°	2 (5.9)	0		1 (4)	0	
IVH 111-IV°	0	1 (1.6)		0	0	

*Reported are median and interquartile range or number and percentage*,

** *Reported p-value over all groups*.

*ARDS, acute respiratory distress syndrome; DIC, disseminated intravascular coagulation; HELLP, haemolysis, elevated liver enzymes, low platelets; IUGR, intrauterine growth restriction; IVH, intraventricular hemorrhage; NEC, necrotising enterocolitis; PE, preeclampsia; wk+d, weeks+days; WOG, weeks of gestation*.

**Table 3:** Diagnostic accuracy in preterm and term PE using different cut off values.

**Preterm PE (*n* = 34)**	**sFit-1:PIGF: Cut offs values ≥85 < 34+0 and ≥110 ≥34+0 WOG**	**sFit-1:PIGF: 95th centile 24–28 WOG ≥10.0 29–33 WOG ≥33.9 34–36 WOG36.4**	**sFit-1: 95th centile 24–28 WOG ≥3205 29–33 WOG ≥5165 34–36 WOG ≥ 7363**	**PIGF: 5th centile 24–28 WOG ≤ 169 29–33 WOG ≤ 114 34–36 WOG ≤ 78**
Sensitivity	0.94 (0.80, 0.99)	1.00 (0.85, 1.00)	1.00 (0.85, 1.00)	0.91 (0.76, 0.98)
Specificity	0.86 (0.75, 0.93)	0.80 (0.68, 0.89)	0.80 (0.68, 0.89)	0.81 (0.70, 0.90)
Term PE (*n* = 25)	sFit-1:PIGF: Cut-off value ≥110	sFit-1:PIGF: 95th centile ≥112	sFit-1: 95th centile ≥9184	PIGF: 5th centile ≤ 54
Sensitivity	0.28 (0.12, 0.49)	0.28 (0.12, 0.49)	0.48 (0.28, 0.69)	0.00 (0.00, 0.20)
Specificity	0.91 (0.75, 0.96)	0.91 (0.79, 0.98)	0.93 (0.82, 0.99)	0.93 (0.82, 0.99)

*Reported are the sensitivity and specificity and the 95th confidence interval in brackets. PE, preeclampsia; PlGF, placental growth factor; sFlt-1, soluble vascular endothelial growth factor receptor-1; WOG, weeks of gestation*.

## Discussion

### Principal findings and comparison with previous studies

Our study showed that the discrimination of term PE vs. its GA-matched high-risk control group reached not the high diagnostic accuracy using the sFlt-1:PlGF ratio (AUROCC of 0.95 (95% CI, 0.88–0.97) compared to the assessment of preterm PE—as was expected. The 95th centile of sFlt-1 with levels ≥9,184 pg/mL showed a modest result with a sensitivity of 0.48, a specificity of 0.93 and an AUROCC of 0.84 (95% CI, 0.74–0.94) for the detection of term PE. High sFlt-1 levels of up to 18.050 pg/mL in women without clinical signs of PE (hypertension and/or proteinuria) decreased the sensitivity in the term group. The slightly better discrimination of sFlt-1 with an AUROCC of 0.84 (vs. PlGF with an AUROCC of 0.68 and sFlt-1:PlGF ratio with an AUROCC of 0.83) was reported before in a study of 49 women with PE and 146 control women ≥34 WOG (20). Higher test performance for the sFlt-1:PlGF ratio was previously shown with a sensitivity of 58% and a higher specificity of 96% (13), but here late onset PE with a wider range for GA of >34 WOG were included and not only term PE >37 WOG, as in our analysis. In a recent study (17), a sFlt-1:PlGF ratio threshold of ≥110 at 36 WOG had a positive predictive value (PPV) of 30% for the development of severe PE in low and high risk nulliparous women. Unfortunately, the screening performances of single biomarkers were not reported, therefore no statement can be made about whether sFlt-1 alone could have reached a better PPV than the sFlt-1:PlGF ratio. In our analysis, PlGF with the 5th centile of ≤ 54 pg/mL, sFlt-1 with the 95th centile ≥9,184 pg/mL and the sFlt-1:PlGF ratio of ≥110 could be used to rule-out term PE with a high specificity of 0.93 for the single biomarkers and 0.91 for the ratio. In the study by Sovio et al. a sFlt-1:PlGF ratio ≤ 38 showed a high negative predictive value (NPV) of 99.2% at 36 WOG for the later development of PE. The authors were able to stratify the unselected nulliparous population into 5% at high risk and 70% at low risk of developing PE.

PlGF as a biomarker of poor placental function, sFlt-1, and sFlt-1:PlGF ratio might not be recommendable for use alone in determining term PE, which should not be surprising, as late onset PE (>34 WOG) seems to be a maternal disease with only a minor placental component (21). Therefore, other biomarkers need to be identified for a large proportion of term PE. A combination of PlGF and sFlt-1 and their ratio together with other potential biomarkers and/or addition of maternal characteristics might be plausible in PE where a big overlap between maternal and placental component exists.

The performance of the PlGF, sFlt-1, and the PlGF: sFlt-1 ratio was high in preterm PE for the prediction of adverse fetal and neonatal outcome (AUROCC of 0.86–0.92). In term PE, the incidence of fetal or neonatal adverse outcomes was too low to make any conclusion (*n* = 8). Zeisler et al. (8) showed that the sFlt-1: PlGF ratio ≤ 38 had a high NPV of 99.3% for fetal adverse events developing within 1 week and the threshold >38 had a positive predictive ratio of 47.5% for the prediction of adverse fetal events at 4 weeks in their validation cohort of 550 women with symptoms suggestive of PE.

In term PE, delivery is still the only available intervention to treat PE. The randomized control HYPITAT trial (22) showed improved maternal outcome after immediate induction of labor compared to expectant management in women with gestational hypertension or mild preeclampsia at 36 to 41 WOG. If clinical signs suspicious for impending PE such as hypertension and/or proteinuria is present at term, induction of labor or planned delivery should be initiated. But if the wish of the future parents is to wait, sFlt-1 and PlGF can rule out PE accurately at term. The tremendously high NPV of the sFlt-1:PlGF ratio ≤ 38 in pregnant women with signs and symptoms of PE—which could not be proven by our study because of the case-control design, but could be indirectly shown by the high specificity of all biomarkers—might further help clinicians in making the decision to induce or delay labor. Thirty-eight of 39 women with a sFlt-1:PlGF ratio above the threshold of ≥85 or ≥110 in the preterm group and all of the 11 pregnant women with the threshold ≥110 in the term group delivered during the following week independent of the development of PE. This is in line with results of a previous study of 616 women with suspected PE, in which 86% of the women ≤ 34 WOG with a sFlt-1:PlGF ratio ≥85 and 15.8% of women with the ratio < 85 delivered during 2 weeks (hazard ratio, 15.2; 95% CI, 8.0–28.7) (23).

### Strengths and limitations

One strength of this study was the very strict inclusion of analyzed biomarker results which limited the analysis to blood samples taken only on the day of PE diagnosis. Unfortunately, a substantial number of women with PE had to be excluded from data analysis for this reason and by the study design itself as a case-control study. Therefore, a major limitation was the resulting small sample size.

### Implications for clinical practice

Our study confirmed the good diagnostic value of the sFlt-1: PlGF ratio in women with preterm PE ≤ 37 weeks of gestation using the proposed cut-off values of ≥85 for ≤ 34 WOG and of ≥110 for >34 WOG. In term PE, a good clinical strategy based on the currently available knowledge could be to rule out PE using the high NPV of the sFlt-1:PlGF ratio ≤ 38 in women who are clinically suspicious for PE but for whom uncertainty regarding the diagnosis still exists (e.g., symptoms of PE without clinical signs or proteinuria without hypertension). In these cases, expectant management could safely be conducted in screen-negative women. Nevertheless, high sFlt-1 levels >95th centile and a sFlt-1:PlGF ratio ≥110 should raise suspicion for imminent term PE. A prospective study should be performed to evaluate if the PPV and the NPV of sFlt-1 alone or in combination with maternal characteristics can further improve diagnostic accuracy and clinical management in term PE.

In summary, we conclude that in term high risk women, sFlt-1:PlGF ratio and sFlt-1 may help to rule out PE.

## Author contributions

OL was principal investigator in Basel and study protocol author, who obtained ethical approval, and drafted this manuscript together with EH. BM was principle investigator in Geneva and revised the manuscript. InH, AK, and SL were responsible for data acquisition and revised the manuscript. AS performed the statistical analysis. MH contributed to the study design and reviewed the manuscript. IrH made important contributions and critically reviewed the content. All authors have given final approval of the version to be published.

### Conflict of interest statement

MH reports being an employee of Roche Diagnostics, holding stock in Roche, having a pending patent related to the sFlt-1 to PlGF or endoglin:PlGF ratio to rule out onset of preeclampsia in pregnant women within a certain time period (PCT/EP2013/063115), holding pending patents related to the dynamic of sFlt-1 or endoglin:PlGF ratio as an indicator for imminent preeclampsia or the HELLP syndrome or both (PCT/EP2012/072157) and the prediction of postpartum HELLP syndrome, postpartum eclampsia, or postpartum preeclampsia (PCT/EP2015/051457). The latter is also a pending patent by OL, who also has acted as a consultant for Roche Diagnostics. The remaining authors declare that the research was conducted in the absence of any commercial or financial relationships that could be construed as a potential conflict of interest.
